# Neoadjuvant chemoradiation therapy application in radical esophagectomy surgery: Safety and feasibility: A descriptive study in Vietnam

**DOI:** 10.1097/MD.0000000000041429

**Published:** 2025-01-31

**Authors:** An Thi Thoai Nguyen, Thang Huy Quoc Dang, Son Ngoc Dang, Thanh Chi Tran, Nghia Trong Doan, Vinh Quoc Nguyen, Cuong Hung Pham

**Affiliations:** a Oncology Department, University of Medicine and Pharmacy, Ho Chi Minh City, Vietnam; b Thoracic and Abdominal Department, Ho Chi Minh City Oncology Hospital, Ho Chi Minh City, Vietnam.

**Keywords:** esophageal cancer, esophagectomy surgery, feasibility, neoadjuvant chemoradiation therapy, safety

## Abstract

Esophageal cancer (EC) ranks as the 7th most prevalent form of cancer and the 6th leading cause of cancer-related mortality globally. Neoadjuvant therapy, encompassing neoadjuvant chemotherapy or chemoradiotherapy, has shown promise in reducing the staging of EC and mitigating the risk of early systemic spread. This study seeks to assess the safety and viability of implementing neoadjuvant chemoradiotherapy (nCRT) in conjunction with radical esophagectomy surgery for Vietnamese patients diagnosed with locally advanced EC. Safety was evaluated based on the incidence of grade ≥3 treatment-related adverse events, while feasibility was assessed through indicators such as pathological complete response, major pathological response, and R0 resection rates. The study analyzed data from 30 patients, following specific inclusion criteria. Baseline characteristics analysis revealed a participant cohort entirely composed of males, wherein 83.3% were identified as smokers, with tumors predominantly located in the middle (46.7%) and lower (53.3%) regions of the thoracic esophagus. The predominance of clinical stages II and III was observed. The nCRT protocol resulted in a substantial reduction in dysphagia score, with a statistically significant *P* < .001. The median duration from the conclusion of radiation treatment to surgery was 62 days, with a median operative time of 302 minutes and a median estimated blood loss of 189 mL. Surgical complications primarily included anastomotic leakage and pneumonia, occurring in 23.3% and 16.7% of cases, respectively. R0 resection was achieved in 29 (96.7%) patients, with 43.4% attaining pathological complete response and 56.7% demonstrating tumor complete response. The study’s outcomes emphasize the safety and feasibility of employing esophagectomy subsequent to nCRT in Vietnamese patients, as evidenced by the absence of mortality, low complication rates, and favorable surgical results. It also suggests the potential advantages of utilizing a lower daily Gy dose for enhanced safety and considering squamous cell carcinoma as a specific criterion for nCRT.

## 1. Introduction

Esophageal cancer (EC) is currently the 7th most common cancer and the 6th leading cause of cancer-related death all over the world.^[1]^ Neoadjuvant therapy including neoadjuvant chemotherapy (nCT) or neoadjuvant chemoradiotherapy (nCRT) has demonstrated potential advantages in downstaging EC and reducing the risk of early systemic dissemination. Advanced EC, categorized as stage IIb to IIIc, represents a significant proportion of EC cases in Vietnam, with squamous cell carcinoma (SCC) being the prevailing histological subtype. The management of this stage remains subject to ongoing debates due to discrepancies among different guidelines. Despite being a traditional approach for resectable EC, esophagectomy is associated with notable challenges, including a heightened risk of recurrence, distant metastasis, compromised survival, and unfavorable prognosis.^[2]^

In Western countries, the survival benefits of nCRT over surgery alone have been established through various clinical trials, notably the nCRT for EC followed by surgery study trials, leading to its acceptance as the standard treatment for locally advanced EC.^[3]^ Although the regimen offered survival benefits for patients with both histologic subtypes, it was more obvious in those with SCC than in those with adenocarcinoma (AC). Overall, nCRT could provide a 3-year survival advantage to nCT, which was mainly reflected in the SCC subtype and patients with stage IV cancer.^[4]^

In Asian countries, physicians often exhibit reluctance in directly adopting these Western findings into their clinical practice. This hesitancy stems from the fact that SCC cases are common in Asia,^[4]^ which require the approach of transthoracic esophagectomy with regional lymphadenectomy can potentially achieve superior local control by nCT.^[5]^ Besides, there is a lack of evidence that demonstrates the superior benefit of nCRT over nCT in the Asia population. Thus, our study aimed to describe the safety and feasibility of applying nCRT in radical esophagectomy surgery in Vietnamese patients diagnosed with locally advanced EC.

## 2. Materials and methods

### 2.1. Study design

The retrospective study was done by reviewing and collecting data from the hospital database of patients in the Department of Abdominal and Thoracic Surgery (Oncology Hospital, Vietnam) from 2020 until December 2022.

### 2.2. Inclusive criteria

- EC stages II to III (T2-3N0-2M0).
- Tumor located at thoracic level (25–40 cm) and confirmed by SCC pathology.
- The Eastern Cooperative Oncology Group performance status scale (0–1).
- Cardiac function: no abnormality and left ventricular ejection fraction > 50%.
- Respiratory function: forced vital capacity and forced expiratory volume 1 ≥ 50%.
- All surgeries were performed by the same surgical team.

### 2.3. Exclusive criteria

- Associated with other malignant diseases.
- Previous history of chemotherapy or radiotherapy.

### 2.4. Neoadjuvant chemoradiation therapy

Patients received weekly administration of 5 cycles of nCRT as follows:

- Five days of intravenous chemotherapy: on days 1, 8, 15, 22, and 29, carboplatin (2 mg/mL/min) followed the area under the curve dose calculation as described in Janowitz et al’s^[6]^ study, and paclitaxel at a dose of 50 mg/m^2^ of body-surface area.^[7]^
- Twenty-three days of radiotherapy (41.4 Gy, given in 23 fractions of 1.8 Gy on 5 d/wk).^[7]^

After a minimum of 4 weeks following completion, all patients underwent reassessment using gastrointestinal endoscopy, cervical, thoracic, and abdominal enhanced computed tomography, in accordance with the response evaluation criteria in solid tumors.

### 2.5. Surgical techniques

McKeown esophagectomy, utilizing a 3-hole approach, was conducted following a minimum 6-week period after the completion of nCRT. The decision to perform a 2-field lymphadenectomy (thoracic and abdominal) or a 3-field lymphadenectomy (cervical, thoracic, and abdominal) was made based on preoperative evaluation. The mobilization of the thoracic esophagus and the performance of mediastinal lymphadenectomy were achieved using the right thoracoscopic approach. Subsequently, reconstruction was carried out by creating a gastric tube and utilizing the posterior mediastinal route. Cervical esophagogastrostomy, either through a hand-sewn double-layer suture or a circular stapler with a diameter of 25 mm, was then performed. Thoracostomy tubes were inserted under direct visualization and remained in place until confirmation of safety through radiographic imaging. In addition, a Penrose drain was placed at the left neck, specifically at the site of the anastomosis, to monitor potential leakage.^[8]^

### 2.6. Safety and feasibility evaluation

Safety was evaluated based on the incidence of grade ≥3 treatment-related adverse events including the operation time, intraoperative blood loss, the number of retrieved lymph nodes, the status of surgical margin resection, the rate of pathological complete response (pCR), and the incidence of perioperative complications. These complications encompassed both anastomosis-related complications and anastomosis-nonrelated complications.

Feasibility was evaluated by the efficacy of treatment via pCR, major pathological response by the RECIST v1.17 criteria, and R0 resection rates which was determined by the absence of cancer cells at both the proximal and distal margin, indicating a negative resection margin.

### 2.7. Other clinical data

Other data collection encompassed a comprehensive set of investigations including clinical examination, esophagogastroduodenoscopy, enhanced computed tomography of the neck, thorax, and abdomen, bronchoscopy, spirometry test, cardiac ultrasound, and perioperative blood tests. In cases where there was uncertainty regarding the tumor stage (T1 or T2), an esophageal endoscopic ultrasound was conducted. In addition, magnetic resonance imaging and/or positron emission tomography were employed to evaluate potential metastases in situations requiring further clarification.

### 2.8. Statistical analysis

All statistical analyses were conducted using SPSS version 20. Continuous variables were presented as medians with interquartile deviation, while categorical variables were displayed as frequencies and percentages. To compare groups, the χ^2^ test, Fisher exact test, Student *t* test, or analysis of variance test was utilized based on the nature of the parameters. A significance level of *P* < .05 was used to determine statistical significance.

## 3. Results

### 3.1. Baseline patient characteristics

A total of 30 patients’ data were obtained from our hospital database based on specific inclusion criteria. Analysis of baseline characteristics showed that all participants were male, with 83.3% identified as smokers and tumor location predominantly situated in the middle and lower sections of the thoracic esophagus at 46.7% and 53.3%, respectively. The majority of clinical stages were observed to be in stages II and III, as indicated in Table 1.

**Table 1:** Patient with EC characteristics at baseline (n = 30).

Characteristics	N (%)
Age (yr), mean ± SD	55 ± 5.1
Male	30 (100)
Smoking	25 (83.3)
Tumor location	
Upper	–
Middle	14 (46.7)
Lower	16 (53.3)
Clinical stage	
T2	3 (10)
T3	27 (90)
N0	15 (50)
N1	12 (40)
N2	3 (10)
Stage II	16 (53.3)
Stage III	14 (46.7)

EC = esophageal cancer, SD = standard deviation.

### 3.2. Patient’s status after nCRT

The primary anticipated outcome of nCRT is the alleviation of dysphagia in patients. Our nCRT protocol demonstrated a notable reduction in dysphagia score, decreasing from 1.96 to 0.43 among patients in the Ogilvie scale, with a statistically significant *P* < .001. At the end of treatment, nearly 60% of the patients were able to eat solid food. Conversely, there were no significant changes observed in patients’ body mass index (BMI) and serum albumin levels following nCRT (*P* > .05), and pulmonary function remained unaffected. Hematologic complications as acute toxicity of nCRT were identified in only 4 (13.3%) patients, while other toxicities such as hepatic failure, esophageal fistula, and esophagitis were not present within our patient cohort (as indicated in Table 2).

**Table 2:** Patients’ status after nCRT.

	Baseline	After nCRT	*P*
BMI	20 ± 2	19 ± 3	.89
Albumin (g/dL)	4.26 ± 0.3	4.04 ± 0.3	.27
Pulmonary function			
FEV1 (L)	2.71 ± 0.2	2.10 ± 0.3	.53
Dysphagia relief (Ogilvie score)	1.96	0.43	<.001
Acute toxicities of nCRT			
Hepatic failure	–	0	–
Esophageal fistula	–	0	–
Esophagitis	–	0	–
Hematologic complications	–	4 (13.3%)	–

BMI = body mass index, FEV1 = forced expiratory volume at 1 second, nCRT = neoadjuvant chemoradiotherapy.

### 3.3. Surgery safety and efficacy

The assessment of surgical safety primarily relies on several key parameters, as detailed in Table 3. The median duration from the conclusion of radiation treatment to surgery was 62 days. Operative time exhibited a median of 302 minutes, while the median estimated blood loss was 189 mL. Notably, surgical complications primarily consisted of anastomotic leakage and pneumonia, with incidences of 23.3% and 16.7%, respectively.

**Table 3:** Surgery safety and efficacy (n = 30).

	N (%)
Surgery parameters	
Interval to surgery (d)	62 ± SD
Operative time (min)	302 ± 66
Average blood loss (mL), (min–max)	189 (50–600)
Surgery complications	
Anastomotic leakage	7 (23.3)
Pneumonia	5 (16.7)
Pleural effusion	3 (10)
Pneumothorax	1 (3.3)
Other	0
Surgery outcomes	
R0	29 (96.7)
Average number of harvested LN	20 ± 9
Average number of positive node, (min–max)	1.1 (0–10)
Tumor completed response	17 (56.7)
pCR	13 (43.4)
Surgery death or readmission	
30-d Mortality	0
30-d Readmission	0

min–max, minimum–maximum, pCR = pathological complete response, SD = standard deviation.

Immediate surgical efficacy was evaluated based on patients’ clinical improvement. Among the 30 surgical patients, R0 resection was accomplished in 29 (96.7%) patients. The average number of harvested lymph nodes was 20, with an average of 1.1 positive nodes. A total of 13 (43.4%) achieved a pCR, and a tumor complete response was observed in 56.7% of cases. Importantly, there were no deaths or readmissions within 30 days following discharge in our study.

## 4. Discussion

In our safety assessment of nCRT in Vietnamese patients with EC, we observed 2 significant safety outcomes for the patients. First, the safety of nCRT was demonstrated by a substantial decrease in dysphagia scores before and after the intervention, with respective values of 1.96 and 0.43. In addition, we found no significant impact on albumin levels, BMI, pulmonary functions, and the only observed complication was related to hematology. Second, the subsequent esophagectomy surgery after nCRT exhibited a high rate of complete pathological response (43.4%), fewer complications, and no mortalities. Our feasibility assessment also considered the operation time and blood loss.

The safety of nCRT has been demonstrated by a significant decrease in dysphagia scores before and after treatment in previous studies, as evidenced by the reduction in Ogilvie score from 2 to 0.65 (*P* < .001)^[9]^ and quality of life in patients with gastric and esophageal cancers (QLQ-OG25) score from 41 to 28 (*P* = .039).^[10]^ Similarly, our study showed a significant decrease in the Ogilvie score from 1.96 to 0.43 (*P* < .001). Furthermore, the concentration of albumin in the blood has been identified as an important factor in patient safety. Our findings indicate that patients with high serum albumin levels at baseline experienced no impact after nCRT. In contrast, patients with low serum albumin levels at baseline showed a significant decrease in concentration from 3.75 to 3.49 (*P* < .001).^[11]^ It is plausible that a low initial concentration may result in a rapid decrease in albumin levels. Based on our results, we recommend the use of high serum albumin levels as a criterion for patient selection to ensure patient safety.

In addition, other factors such as BMI and pulmonary functions exhibited only minor fluctuations in response to nCRT in our study, in accordance with the chemoradiotherapy for esophageal cancer followed by surgery study (CROSS) protocol.^[7]^ Regarding patients’ BMI, our findings align with those of Sunde et al’s^[10]^ study, which reported no significant change following nCRT. Conversely, pulmonary function was notably impacted in a study by Chen et al,^[12]^ demonstrating a decrease in forced expiratory volume at 1 second from 2.66 to 2.18 L (*P* = .023). We observed a disparity in the daily radiation dose between our study and that of Chen et al’s study (1.8 vs 2 Gy daily), which may account for the adverse effect on pulmonary function associated with the higher daily Gy dose. Therefore, it is advisable to employ a lower daily Gy dose to safeguard pulmonary function and minimize complications. We encountered only hematologic complications (13.3%), which were manageable. Notably, there were no mortalities in our cohort following surgery, consistent with findings from previous studies.^[13]^

In numerous studies, the safety of esophagectomy surgery following nCRT has been a focal point, affirming the viability of this innovative procedure. Within our study, the median operating time aligned closely with that of other investigations, at 302 minutes compared with the range of 253–419 minutes.^[14,15]^ The acceptable blood loss in general surgery varies by age and sex and is determined using the formula for allowable blood loss (ABL), which is calculated as “ABL = estimated blood volume (EBV) × initial hematocrit (Hi) – final hematocrit/Hi,” following 2 steps: calculation of EBV, where EBV = weight (kg) × blood volume (age and gender) and determination of the tolerated decrease in hematocrit from the Hi by the patient.^[16]^ Our cohort consisted entirely of males with an average weight of 60 kg, resulting in an acceptable ABL of 350 mL. With our average blood loss during surgery amounting to 189 mL, it can be inferred that the procedure was deemed safe as it remained below the acceptable ABL.

Postoperative pneumonia constitutes a significant complication subsequent to esophagectomy, with reported incidence rates ranging from 20% to 40%.^[17]^ For instance, in the study by Chen et al,^[12]^ the occurrence rate was 13.51%, whereas our investigation revealed a lower rate of 8.3%. This disparity may be attributed to the differences in daily radiation dosage applied, as evidenced by our utilization of a lower daily dose of 1.8 Gy compared with the standard 2 Gy regimen. The incidence of anastomosis leak in our study was 23.3%, aligning with prior research findings ranging from 20.3% to 40%.^[18,19]^

In our cohort, nearly half of the patients (43.4%) exhibited a pCR, surpassing the 26% rate observed in the CROSS trial. This outcome can be attributed to our rigorous patient selection criteria, focusing on the histology of SCC, which has been associated with a higher sensitivity to radiation therapy compared with adenocarcinoma (49% vs 23%).^[7]^ In addition, the primary tumor response rate in our study was 56.7%, akin to rates observed in Asian (33.8%) and Dutch (50.4%) patient populations.^[20]^

This study is subject to several limitations, including a restricted patient population size stemming from the scarcity of individuals meeting our stringent selection criteria, a common challenge encountered by similar studies; and its retrospective nature, lacking randomization and thereby resulting in less robust and representative findings.

## 5. Conclusion

The results of our study demonstrate the safety and feasibility of employing esophagectomy following nCRT in Vietnamese patients, as evidenced by the absence of mortality, low incidence of complications, and favorable surgical outcomes. Our findings suggest the potential benefits of employing a lower daily Gy dose for enhanced safety, as well as the selective consideration of SCC as a specific criterion for nCRT.

## Acknowledgments

The authors would like to thank Ho Chi Minh City Oncology Hospital for supporting us to conduct this research.

## Author contributions

**Conceptualization:** An Thi Thoai Nguyen, Thanh Chi Tran, Nghia Trong Doan.

**Data curation:** An Thi Thoai Nguyen, Thang Huy Quoc Dang, Son Ngoc Dang, Vinh Quoc Nguyen, Cuong Hung Pham.

**Formal analysis:** An Thi Thoai Nguyen, Thang Huy Quoc Dang, Nghia Trong Doan, Cuong Hung Pham.

**Investigation:** An Thi Thoai Nguyen, Son Ngoc Dang, Vinh Quoc Nguyen, Cuong Hung Pham.

**Methodology:** An Thi Thoai Nguyen, Thang Huy Quoc Dang, Son Ngoc Dang, Nghia Trong Doan, Vinh Quoc Nguyen, Cuong Hung Pham.

**Project administration:** An Thi Thoai Nguyen.

**Writing – original draft:** An Thi Thoai Nguyen.

**Supervision:** Thang Huy Quoc Dang, Thanh Chi Tran, Nghia Trong Doan, Vinh Quoc Nguyen, Cuong Hung Pham.

**Validation:** Son Ngoc Dang, Thanh Chi Tran, Nghia Trong Doan.

**Writing – review & editing:** Thanh Chi Tran.
